# Evaluation of the Efficacy of Corticotomy and Piezocision on Canine Retraction: A Systematic Review

**DOI:** 10.3390/dj13020057

**Published:** 2025-01-27

**Authors:** Erica Lipani, Elisa Pisani, Mariagrazia Verrone, Federica Bitonto, Alessio Verdecchia, Enrico Spinas

**Affiliations:** Department of Surgical Sciences, Orthodontics School, University of Cagliari, Via Ospedale, 09124 Cagliari, Italy; elisapisani@hotmail.it (E.P.); mariagrazia.odonto@gmail.com (M.V.); federibit@gmail.com (F.B.); verdecchia.belli.a@gmail.com (A.V.)

**Keywords:** corticotomy, piezocision, regional acceleratory phenomenon, canine retraction

## Abstract

**Background:** In order to reduce the prolonged duration of orthodontic treatment, several surgical techniques have been proposed over the years. Corticotomy and piezocision are the two most widely used techniques, and, given the lack of consensus in the literature, along with the renewed interest in these approaches in recent years, the primary objective of this study is to evaluate their effectiveness in accelerating canine retraction in patients requiring extraction of the upper first premolar and, as a secondary objective, to assess if there is a worsening of periodontal health and how the surgical approach is perceived by the patient. **Methods:** An electronic search was performed on PubMed, Scopus, Web of Science, Embase, and the Cochrane Central Register of Controlled Trials (CENTRAL) up to 30 November 2024. The PRISMA statement was adopted for the realization of the review, and the Cochrane Collaboration’s risk of bias assessment tool (RoB 2) was used to assess the studies’ quality. **Results:** After full text assessment, fifteen randomized clinical trials (14 split mouth design, 1 single-blind, single-center design) covering 326 patients (mean age 20, 19 years) were included. The data collected reveal that corticotomy accelerates canine retraction by 1.5 to 4 times, while piezocision achieves retraction 1.5 to 2 times faster, making corticotomy the most effective technique. No statistically significant adverse effects on periodontal ligament, molar anchorage loss, or root resorption were observed following the two surgical techniques. In addition, patients reported experiencing mild to moderate pain. **Conclusions:** Corticotomy and piezocision are effective techniques for accelerating upper canine retraction in extraction cases, significantly reducing the overall duration of orthodontic treatment.

## 1. Introduction

The long duration of orthodontic treatment is one of the major concerns for orthodontic patients, especially given the increased proportion of adults seeking orthodontic treatment. Several factors influence the length of therapy, including age, gender, arch, and bone quality [1,2,3,4]. Moreover, shortening the duration of the treatment is not only satisfying for the patient, but it may also minimize and reduce the risk of experiencing various adverse effects such as pain, discomfort, caries, white spot appearance, gingival recession, and apical root resorption associated with the orthodontic therapy [5].

Over the years, several techniques have been proposed to decrease the overall orthodontic treatment time, and they can be classified into three categories:

(1) Pharmacological: vitamin D3 [6], corticosteroids [7], and prostaglandins [8] administered locally and systemically;
(2) Physical: local heat application, low-level laser therapy (LLLT) [9], light-emitting diode (LED) therapy [10], and light vibrational forces (LVF) [11];
(3) Surgical: corticotomy, corticision, and piezocision.

Among them, the surgical approaches have attracted considerable scientific interest.

Corticotomy is the surgical procedure consisting of intentional cutting using surgical burs of the cortical bone in order to remove the resistance of dense cortical bone.

What happens in bone structures consequently to a corticotomy was first explained by Frost, who introduced the concept of regional acceleratory phenomenon (RAP) [12]. After surgical bone injury, there is a local transitory acceleration in bone turnover activity, and consequently, as a result, there is a reduction in bone density, which is expected to facilitate orthodontic dental movement [13]. However, it remains an invasive technique requiring flap elevation, causing postoperative discomfort and posing potential complications, and therefore is often rejected by patients.

Over time, alternative, less invasive techniques have been introduced aiming to overcome these complications. One of the methods proposed to respond to this need was corticision; this surgical procedure does not require flap elevation and is performed using a reinforced scalpel and a mallet [14]. However, the existing literature on corticision primarily relies on studies conducted with animals [15,16].

Thereafter, based on the corticision technique, a new technique called piezocision was proposed, characterized by the replacement of the scalpel and mallet with a piezosurgical knife. First described by Dibart et al., it consists of microincisions in the gingival tissue and bone incisions made with this ultrasonic piezosurgical knife [17]. This is a safer technique without the fear of damaging the tooth root and with excellent control of bleeding. Furthermore, selective tunneling can be performed when soft or hard tissue grafting is required [18].

The application of these surgical techniques has been proposed and used to accelerate several stages of orthodontic therapy; among them, space closure and canine retraction are two of the most challenging and time-consuming treatment steps. This is significant considering that Class II malocclusion is the most common malocclusion, often resolved by extracting two premolars. Moreover, extraction of the upper bicuspids is commonly performed to resolve class I cases with biprotrusion or increased overjet.

Referring to the timing, an average of 5 months is needed to achieve complete maxillary canine retraction with fixed orthodontic appliances, recording a total duration of therapy of approximately 1.5 to 2 years with fixed appliances [19].

Considering that corticotomy and piezocision are the two techniques that have been most widely employed, that no consensus exists yet in the literature, and that mainly in recent years an interest in these two techniques has revived, the main purpose of the present study is to assess if the integration of these surgical techniques will make traditional orthodontic therapy more efficient in patients requiring maxillary first premolar extraction. In addition, an attempt was made to determine whether, as a second objective, a deterioration of periodontal health occurs and how the surgical approach is experienced by the patient.

## 2. Materials and Methods

The present systematic review was performed in accordance with the PRISMA statement of Preferred Reporting Items for Systematic Reviews and Meta-Analyses (PRISMA) [20]. The protocol was registered in PROSPERO, the international prospective register of systematic reviews, under the number CRD42024615824.

### 2.1. Eligibility Criteria

Inclusion and exclusion criteria were established according to the PICO framework, defined as follows: “(P) in patients requiring maxillary first premolar extraction and subsequent canine retraction (I) could corticotomy or (C) piezocision (O) accelerate orthodontic movement?”.

The included studies were randomized clinical trials (RCTs) concerning the maxillary canine retraction in young or adult healthy patients treated using fixed multibracket appliances with corticotomy and/or the piezocision procedure; articles reporting the duration of treatment, speed, and/or rate of tooth movement with corticotomy and/or the piezocision procedure compared to conventional orthodontic treatment.

The excluded studies were the ones on patients with previous orthodontic treatment, systemic diseases, periodontal disease, or any disorders or therapies that might have affected bone turnover or density. Case-control, cohort, and cross-sectional, non-randomized clinical trials (Nr-RCTs); reviews; case reports; case series; in vitro; and animal studies were rejected. Inclusion and exclusion criteria are also summarized in Table 1.

### 2.2. Information Sources and Search Strategy

A comprehensive search strategy was designed, and the following online databases were recruited for the search: PubMed, Scopus, Web of Science, Embase, and the Cochrane Central Register of Controlled Trials (CENTRAL). The search was conducted until 30 November 2024, and no time and language restrictions were applied.

For all other databases, the same advanced search was performed using the same combination of keywords, MeSH (medical subject heading) terms, and Boolean operators. The strategies designed for each database are reported in Table 2.

### 2.3. Selection Process 

Two authors (E.L. and E.P.) independently conducted first the research process and subsequently the screening of the obtained results. To evaluate the agreement level among the reviewers, Cohen’s kappa coefficient [21] was calculated, and, in case of disagreement, a third reviewer was consulted (E.S.). Authors showed a substantial agreement (Cohen’s kappa: 0.66). Therefore, an initial screening was conducted based on titles and abstracts, selecting all potentially eligible studies that focused on canine retraction.

Subsequently, a second screening was conducted on the full text of the articles according to the established inclusion and exclusion criteria.

### 2.4. Data Collection

The two main reviewers selected and extracted the data of interest according to the Cochrane Consumers and Communication Review Group data extraction model.

The following data were extracted from the selected papers:

(a) Authors and year of publication;
(b) Study type;
(c) Sample size of study group and of control group;
(d) Gender of participants;
(e) Average age;
(f) Characteristics of malocclusion;
(g) Surgical intervention;
(h) Type of orthodontic fixed appliances;
(i) Force reactivation;
(j) Results.

The primary outcome registered was the rate/velocity of canine retraction data; data on anchorage loss, canine root resorption, periodontal indexes, and the patient’s experience were considered as a secondary outcome.

### 2.5. Quality Assessment

Articles included in the review underwent a quality assessment; the risk of bias of the included randomized clinical trial was independently conducted by two authors (E.L. and F.B.), using the Cochrane Collaboration’s risk of bias assessment tool (RoB 2) [22].

Any disagreement was discussed and resolved by consensus when necessary.

Bias is assessed in five distinct domains: the randomization process, deviations from intended interventions, missing outcome data, measurement of the outcome, and selection of the reported result. Finally, all included studies were evaluated as low risk, some concerns, or high risk.

## 3. Results

### 3.1. Study Selection

**Figure 1:**
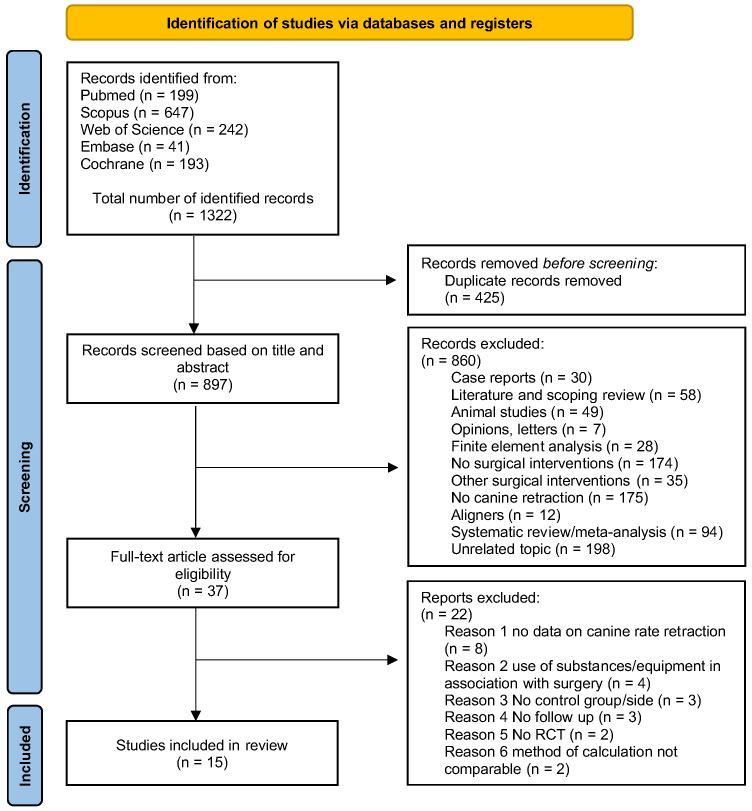
PRISMA flowchart, flow diagram of the performed search.

The database search retrieved 1322 abstracts: 199 from PubMed, 647 from Scopus, 242 from Web of Science, and 193 from Cochrane. Following duplicate removal, 897 papers were screened through the reading of the title and abstract and excluded according to the inclusion and exclusion criteria. After this initial screening, 37 studies were assessed for eligibility based on reading the full text. Finally, 15 RCTs were included in the qualitative synthesis. The flowchart of the screening process according to the PRISMA statement is shown in Figure 1. 

The comprehensive PRISMA 2020 Checklist, outlining all reporting elements addressed in this review, is available in Table S1 in the Supplementary Material.

### 3.2. Study Characteristics

The key characteristics of the included studies are shown in Table 3. All included studies were RCTs, all of them with a split-mouth design [23,24,25,26,27,28,29,30,31,32,33,34,35,36] except for one with a single-blind, single-center design [37]. All included studies evaluated the efficacy of corticotomy and/or piezocision compared to conventional orthodontic treatment in upper canine retraction after upper first premolar extraction. Concretely, five of them compared conventional canine retraction with piezocision [25,28,30,31,33], seven with corticotomy [24,27,29,32,34,35,36,37], and in three studies both surgical techniques were evaluated [23,26]. The detailed description of the surgical technique can be found in Table S2 in the Supplementary Material.

#### 3.2.1. Surgical Technique

The surgical technique employed in each trial is detailed in Table 3.

Corticotomy was performed in ten studies [23,24,27,29,32,34,35,36,37]. In five of these studies [23,24,32,34,35], only a buccal flap was performed; in two [27,37], both buccal and palatal flaps were elevated; and in three, no flap was raised [26,29,36].

In studies involving corticotomy, a certain variability was observed in the surgical techniques employed as well as in the equipment used. Specifically, some studies performed corticotomy with vertical cuts [23,37], while others utilized perforations [24,26,29,36], and several combined both approaches [27,32,34,35]. In the majority of studies, round burs mounted on a micromotor were utilized [24,27,32,35,36], while a piezotome was employed in two studies [23,37], although Er:YAG laser technology was used in corticotomy flapless procedures [26,27,36].

Piezocision was performed in seven studies [23,25,26,28,30,31,33] and, in all cases, the surgery was flapless, following a similar surgical technique. Notably, in all studies, the procedure was performed using a piezotome to create vertical incisions.

Regarding the timing of extraction, in four studies, the surgical procedure was performed immediately after premolar extraction [23,24,32,35]. In three studies, this information was not clearly provided [25,33,34]; however, in most cases, premolar extractions were carried out significantly earlier than the piezocision and/or corticotomy, ranging from four weeks [27] to four months prior [29,30], or even before the orthodontic appliance was applied [26,28,31,36,37].

#### 3.2.2. Orthodontic Appliance

Properties relating to the orthodontic appliances, canine traction systems, and anchorage methods employed in each trial were described in Table 3.

In the majority of the included studies, the application of orthodontic force for canine retraction occurred during the same appointment in which piezocision and/or corticotomy were performed. Exceptions were found in three studies: in two of them [34,37], force application started three days later, while in the third it was not started until two weeks later [32]. However, in all studies, the alignment and leveling stages were first completed, and only after the insertion of a rigid steel wire did the canine retraction begin. 

Canine retraction was achieved in all studies using a closed NiTi coil spring with an average force of 150 g (ranging from 120 to 250 gr). Only two studies diverged: in one, an elastic chain was used [25], while in the other, an open vertical loop was applied [32]; however, in both cases, the force exerted was comparable to that used in the other studies.

### 3.3. Canine Rate Retraction

The primary outcome assessed was the amount of canine distalization, and the values reported in each study are summarized in Table 3.

In six out of seven studies, canine retraction was significantly faster on the side/group where piezocision was performed than in the control side/group [23,25,26,28,30,33]. Notably, this finding was consistently significant during the first two months of observation. Only one study did not find a statistically significant difference [31] (*p* > 0.05).

Canine retraction was significantly faster in the side or corticotomy group than in the control in 9 out of 10 studies [23,24,27,32,34,35,36,37]. A statistically significant difference was not observed in one single study [29] (*p* > 0.05).

Two studies assessed both surgical techniques [23,26]: Abbas [23] reported that corticotomy produced a canine retraction 1.5 to 2 times faster, while piezocision accelerated retraction by 1.5 times. Consequently, corticotomy produced higher rates of canine movement than piezocision. In contrast, Alfawal’s study [26] found no statistically significant differences between the two surgical techniques (*p* > 0.05).

### 3.4. Risk of Bias Assessment

**Figure 2:**
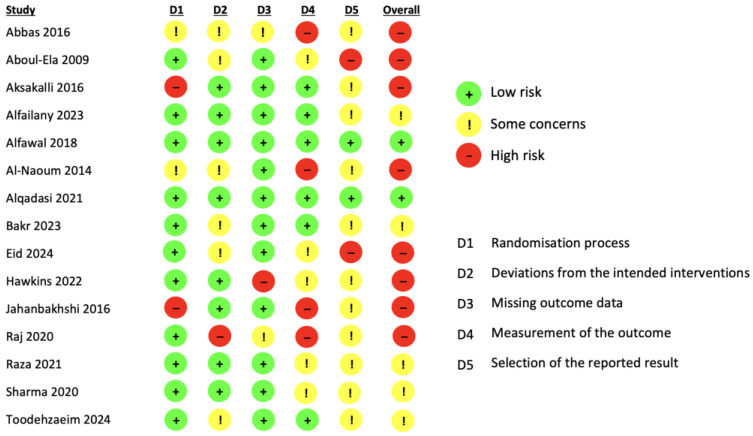
Risk of bias of the included studies according to the RoB 2, Cochrane Collaboration’s risk of bias assessment tool [23,24,25,26,27,28,29,30,31,32,33,34,35,36,37].

According to RoB 2 (Cochrane Collaboration’s risk of bias assessment tool), the quality of the included studies is described in Figure 2. Eight studies were categorized as having a high risk of bias [23,24,25,27,30,31,32,33], five studies were evaluated with some concerns [29,34,35,36,37] and only two studies were classified as low risk [26,28].

A high risk of bias is unfortunately common in orthodontic studies, particularly in those using a split-mouth design. This is primarily because the nature of the interventions makes it impossible to blind patients, operators, or outcome assessors, which inherently increases the risk of performance and detection bias. Furthermore, the independence of outcomes and site-level randomization require careful consideration to avoid inflated assessments of the risk of bias.

## 4. Discussion

Based on the results of the present systematic review, it can be deduced that both corticotomy and piezocision are able to effectively improve the rate of canine retraction following the extraction of the maxillary first premolar compared to conventional orthodontic therapy. Indeed, the amount of canine retraction (primary outcome) achieved with corticotomy was 1.5 to 4 times greater compared to conventional therapy [23,24,27,32,34,35,36,37]. In a comparable manner, piezocision also resulted in a higher rate of canine retraction, ranging from 1.5 to 2 times faster compared to conventional orthodontics [23,25,26,28,30,33]. The measurement of canine retraction was performed directly in the oral cavity using a digital caliper in three studies [27,32,33]. However, in the majority of studies, an indirect measurement method was employed, as described by Ziegler and Ingervall [38].

Specifically, in seven studies, measurements were conducted digitally on plaster models that were subsequently scanned [23,24,25,28,29,31,36]; in two studies, measurements were performed directly on plaster models [34,37]; in another two studies, measurements were performed on photographs of dental models, and a millimeter ruler was included in the images to serve as a reference for correcting magnification and ensuring accurate linear measurements.

In extractive orthodontic cases, anchorage management is particularly critical, for which reason additional anchorage methods, such as miniscrews [24,28,29,30,32,35] or transpalatal arches [26,27,31,33,34,36,37], were used in almost all studies; it is noteworthy that the use of mini-implants for skeletal anchorage is becoming increasingly common [39]. Specifically, molar anchorage loss has been a finding of interest in the selected studies; in several studies there were no differences in molar anchorage loss between the surgical and the control side/group (*p* > 0.05) [23,24,26,31,35,36]. However, a greater loss of anchorage was recorded in the control group/side in three studies [25,29,37], showing that surgical interventions do not affect anchorage stability.

Root resorption is often considered to be the most regrettable complication of orthodontic treatment. The topic has yielded conflicting results in the literature; indeed, in terms of canine root resorption, the control groups of two studies showed a greater resorption [23,34], confirming the findings of Frost [12] that higher osteoclast activity and lower bone density linked to RAP osteopenia reduced the risk of root resorption. This suggested that surgical techniques succeed in accelerating tooth movement without nevertheless inducing root resorption. However, two studies [28,29] did not report significant differences in root resorption, whereas Eid et al. [30] described a greater root resorption, observed concomitantly with piezocision. Only one study used bone grafting in combination with corticotomy [34]; bone grafting would produce an increase in bone formation after an initial phase of bone turnover at the graft site [40].

Historically, corticotomy has been associated with a detrimental effect on the periodontal structures, leading to bone loss and gingival attachment loss. Periodontal status was assessed in seven studies [23,24,25,29,33,35,36], determined by considering parameters like the plaque index, the gingival index according to Silness and Loe’s method, probing depth, attachment level, and gingival recession.

The findings reported in the included articles appear encouraging, considering that the majority of studies did not report significant adverse periodontal effects [23,25,33,35,36]; this suggests that surgical interventions do not negatively impact periodontal health; however, careful periodontal monitoring post-surgery remains essential.

Aboul-Ela et al. [24] reported a significantly higher gingival index in the surgical group, while Bakr et al. [29] observed a significant increase in the plaque index in the group undergoing the surgical technique. Nevertheless, these findings are not considered clinically relevant due to their reversible nature. Excessive tipping or torquing movements in thin biotypes can lead to alveolar bone resorption, while controlled forces can stimulate adaptive remodeling without compromising periodontal health [41]. Adjunctive surgical techniques, such as piezocision and corticotomy, offer potential benefits in managing periodontal responses during orthodontic treatment. By accelerating tooth movement and reducing treatment duration, these approaches may help mitigate prolonged stress on the periodontium, which is particularly advantageous for thin biotypes prone to complications.

In the collective imagination, surgical procedures are often associated with pain and discomfort; however, in a case like this, where premolar extraction is necessary regardless, does the addition of corticotomy and/or piezocision lead to a notable increase in these sensations? Patient comfort and pain level have become topics of increasing interest in recent years, and, indeed, it is an aspect that has been measured in the five most recent studies [27,31,34,35,36]. In four studies, mild to moderate pain, discomfort, and swelling were recorded on the side where the piezotomy [31] or corticotomy [27,34,35] was performed. Pain, particularly significant on the day of the procedure, progressively decreased over time; for this reason, most patients considered the surgical procedure tolerable and recommended it considering the benefit. No significant differences were found in the pain score between the two groups in one study [36].

Orthodontic treatment involving extractions has long been debated for its potential impact on facial aesthetics. Unfortunately, no studies currently provide a comprehensive evaluation of the facial aesthetics of patients who have undergone extractions. However, this does not represent a significant concern, as the current literature suggests that accurate orthodontic treatment planning with precise control of incisor positioning in extraction and non-extraction cases can mitigate adverse effects on the profile [42].

Numerous RCTs in the literature have investigated the surgical techniques of interest; however, many of these were excluded from the current review as they focused on the efficacy of corticotomy and/or piezocision in en-masse extractive space [43,44] or in the management of impacted canines [45] and third molars [46,47,48]. The results recorded are consistent with those reported in the previous works of Patterson et al. [49] and Han et al. [50]. However, several limitations were noted in both studies: the quality of both papers was considered low due to the presence of multiple methodological problems, high risk of bias, and heterogeneity among the included articles. For example, Patterson’s review included studies on impacted canines and en masse canine retraction. In both cases, insufficient attention was placed on surgical techniques, as studies involving micro-osteoperations were also included without adequate differentiation. In the present review, considerable efforts were made to analyze the surgical techniques in detail and to ensure the comparability of the reported results. We also believe that an extremely optimistic assessment of the risk of bias of the included studies was made in the Han et al. study [50]. In the first month of canine retraction, anchorage loss and canine rotation were significantly lesser in the TC and FCAPs groups than in the control group (*p* < 0.001). On the contrary, the canines’ rotation amount after the completion of retraction was greater in the TC group than in the other two groups (*p* < 0.001).

Furthermore, the included studies were all RCTs with a split-mouth design that centered on canine retraction, with the exception of one study [37], which was a three-arm randomized controlled clinical trial. It is noteworthy how prevalently the split-mouth design is utilized in the orthodontic literature. This study design facilitates the control of inter-individual variability by using each subject as its own control, thus minimizing confounding factors such as age, gender, or baseline characteristics that could potentially influence the results. Moreover, it allows the use of smaller sample sizes, reducing the number of subjects required to reach statistical significance [51]. However, it is important to consider the disadvantages of this study design, such as anatomical variability, that may introduce a potential bias in the results, or ethical concerns, particularly if one of the two treatments is perceived to be less favorable. In addition, these studies generally require a longer follow-up period to monitor the effects of the treatments, which may pose problems in terms of patient compliance, especially for younger patients or those requiring extensive orthodontic procedures.

The present study exhibits several limitations: First, the sample included various dissimilarities: in size, ranging from 10 to 36 participants, but especially in terms of age, which ranged from 15 to 30 years. In patients still growing, RAP phenomena may behave differently.

Second, the follow-up periods, which ranged from 3 to 8 months, considering the type of study, are not long enough, which made it difficult to ensure that no further long-term complications were discovered. Furthermore, it is important to note that the surgical techniques presented in the included studies exhibit considerable variability. Despite these limitations, the positive results found in this study should be recognized for their potential benefits in clinical practice.

Future efforts should focus on conducting studies with larger and more homogeneous samples; follow-up periods should be extended over time, and it would be valuable to gather more information not only on the canine retraction phase but also on the total duration of the treatment.

Another effort should be directed toward the standardization of the surgical technique, as several variables currently exist. Additionally, the integration of digital planning tools, such as 3D imaging and artificial intelligence, into surgical workflows could enhance accuracy and reproducibility. Research could explore the impact of these technologies on patient outcomes and the standardization of procedures.

It would also be highly interesting for future research to focus on developing predictive models that account for patient-specific variables (e.g., bone density, age, or periodontal biotype) to personalize treatments and improve the predictability of outcomes.

## 5. Conclusions

Corticotomy and piezocision could effectively accelerate the retraction of the upper canines in extraction cases, resulting in a reduction in the overall duration of orthodontic treatment.

- Corticotomy has been shown to be slightly more effective, with a rate of 1.5 to 4 times higher than conventional therapy.
- Piezocision results in canine retraction that is 1.5 to 2 times faster than conventional treatment.
- The two surgical techniques studied did not show statistically significant adverse effects on the periodontal ligament, molar anchorage loss, or root resorption.
- Patients report experiencing mild to moderate pain, which is nonetheless regarded as tolerable.

## Figures and Tables

**Table 1:** Inclusion and exclusion criteria.

PICOS	Inclusion Criteria	Exclusion Criteria
Participant	Orthodontic permanent dentition patients requiring first premolar extraction and consequent maxillary canine retraction	Patients with periodontal disease or any disorders/therapies that might have affected bone turnover or density, systematic diseases, previous orthodontic treatment, no first premolar extraction
Intervention	Fixed orthodontic appliance with corticotomy and/or piezocision procedures	Orthognathic surgery, osteogenic distraction, no surgical procedures
Comparison	Fixed orthodontics appliance only	Aligners or any removable appliances
Outcome	Duration of treatment, speed, and/or rate of tooth movement	/
Study design	RCTs ^1^	Case-control, cohort, cross-sectional, Nr-RCTs ^2^; reviews; case reports; case series; in vitro; and animal studies

Inclusion and exclusion criteria were established according to the stated PICO. Abbreviations: ^1^ RCTs, randomized clinical trials; ^2^ Nr-RCTs, non-randomized clinical trials.

**Table 2:** Search strategy for each database.

Database	Search Strategy
PubMed	(((((((((((((corticotomy) OR (piezocision)) OR (piezosurg*)) OR (corticotomy facilitated orthod*)) OR (regional acceleratory phenomenon)) OR (periodontally accelerated osteogenic orthod*)) OR (piezotomy)) OR (piezopuncture)) OR (corticision)) OR (Wilckodontics)) AND (tooth movement)) OR (rate tooth movement)) AND (canine retraction)) AND (orthodontic*)
Scopus	(ALL (corticotomy) OR ALL (piezocision) OR ALL (piezosurgery) OR ALL (corticotomy AND facilitated AND orthod*) OR ALL (regional AND acceleratory AND phenomenon) OR ALL (periodontally AND accelerated AND osteogenic AND orthod*) OR ALL (piezotomy) OR ALL (piezopuncture) OR ALL (corticision) OR ALL (wilckodontics) AND ALL (tooth AND movement) OR ALL (rate AND tooth AND movement) AND ALL (canine AND retraction) AND ALL (orthodontics))
Web of Science	(((((((((((((ALL = (corticotomy)) OR ALL = (piezocision)) OR ALL = (piezosurg*)) OR ALL = (corticotomy facilitated orthod*)) OR ALL = (regional acceleratory phenomenon)) OR ALL = (periodontally accelerated osteogenic orthod*)) OR ALL = (piezotomy)) OR ALL = (piezopuncture)) OR ALL = (corticision)) OR ALL = (Wilckodontics)) AND ALL = (tooth movement)) OR ALL = (rate tooth movement)) AND ALL = (canine retraction)) AND ALL = (orthodontic*)
Embase	((‘corticotomy’/exp OR ‘corticotomy’ OR ‘piezocision’/exp OR ‘piezocision’ OR ‘piezosurgery’/exp OR ‘piezosurgery’ OR ‘corticotomy facilitated orthod* ’ OR ‘regional acceleratory phenomenon’ OR ‘periodontally accelerated osteogenic orthodontics’/exp OR ‘periodontally accelerated osteogenic orthodontics’ OR ‘piezotome’/exp OR ‘piezotome’ OR ‘piezopuncture’ OR ‘corticision’ OR ‘wilckodontics’) AND (‘tooth movement’/exp OR ‘tooth movement’) OR ‘ate tooth movement’) AND (‘canine retraction’/exp OR ‘canine retraction’) AND (‘orthodontics’/exp OR ‘orthodontics’)\
Cochrane Central Register of Controlled Trials	corticotomy in All Text OR piezocision in All Text AND rate tooth movement in All Text AND canine retraction in All Text AND orthodontic in All Text

Detailed descriptions are given of the search conducted in the five selected databases: PubMed, Scopus, Web of Science, Embase, and the Cochrane Central Register of Controlled Trials (CENTRAL). The search was customized to each database.

**Table 3:** Study characteristics and results. Author and year of publication, type of study, intervention (piezotomy and/or corticocision), number of participants and gender, mean age, initial malocclusion, characteristics of the fixed orthodontic appliance, force reactivation, primary outcome results, secondary outcomes, and the follow-up period of the 15 included studies have been compiled in the table.

Authors and Year	Type of Article	Intervention	Participants	Mean Age (Year)	Initial Malocclusion	Orthodontic Fixed Appliance	Force Reactivation	Primary Outcome	Secondary Outcome	Follow-Up
Abbas et al. (2016) [23]	RCT, split-mouth design	C, P	20 patients (gender N/A) 10 C 10 P	15 to 25	Class II\1	0.016 × 0.022 SSW (SW Roth) NiTi CLS (150 gr)	Every 2 weeks	CR is 1.5 to 2 times faster on C-side than CG-side. CR is 1.5 times faster on P-side than CG-side.	- MAL: no differences (*p* > 0.05) - PD: N/A - PH: no differences (*p* > 0.05) - RS: CG- side exhibit a greater canine root resorption than C- and P-side	3 months
Aboul-Ela et al. (2011) [24]	RCT, split-mouth design	C	13 patients (5 M, 8 F)	19	Class II\1	0.016 × 0.022 SSW NiTi CLS (150 gr) from miniscrew (U6–U7)	Every 4 weeks	CR is 2 times faster on C-side than CG-side in the first 2 months, declining to 1.6 the 3rd month, and 1.06 by the end of the 4th month.	- MAL: no differences (*p* > 0.05) - PD: N/A - PH: no differences (*p* > 0.05), except for the gingival index higher (*p* < 0.05) CG-side - RS: N/A	4 months
Aksakalli et al. (2016) [25]	RCT, split-mouth design	P	10 patients (4 M, 6 F)	16.3 ± 2.4	Class II	0.016 × 0.022 SSW (SW Roth) Elastomeric chains (150 gr)	Every 2 weeks	CR is 2 times faster on P-side than CG-side.	- MAL: lesser in P-side. - PD: N/A - PH: no differences (*p* > 0.05) - RS: N/A	3.5 months
Alfailany et al. (2023) [37]	RCT, single-blinded, single-center	C	34 patients (13M, 21 F) CTG: 17 (6M,11F) CG: 17(7M, 10 F)	20.98 ± 1.95 CTG: 21.23 ± 2.33 CG: 20.62 ± 1.48	Class II\1	0.019 × 0.025 SSW (SW MBT) NiTi CLS (150 gr) Transpalatal arch	Every 2 weeks	CR is significantly faster in CTG than CG until the end of the 2nd month.	- MAL: CTG showed less MAL than CG - PD: N/A - PH: N/A - RS: N/A	4 months
Alfawal et al. (2018) [26]	RCT, split-mouth design	C, P	36 patients (24M, 12 F) 18 C 18 P	18.08 ± 3.5C	Class II\1	0.019 × 0.025 SSW (SW MBT) NiTi CLS (150 gr) Transpalatal arch	Every 2 weeks	CR was significantly higher on the C- and P-side than CG-side during the first 2 months.	- MAL: no differences (*p* > 0.05) - PD: N/A - PH: N/A - RS: N/A	4 months
Al-Naoum et al. (2014) [27]	RCT, split-mouth design	C	30 patients (15 M, 15 F)	20.04 ± 3.63	Class II\1 and 2	0.019 × 0.025 SSW (SW MBT) NiTi CLS (120 gr) Transpalatal arch	Every 2 weeks	CR is 4 times faster on C-side than CG-side at 0–2 wks, is 3 times faster at 4 and 12 wks.	- MAL: N/A - PD: >50% of the sample had moderate to severe discomfort on the surgical side - PH: N/A - RS: N/A	3 months
Alqadasi et al. (2021) [28]	RCT, split-mouth design	P	11 patients (5 M, 6 F)	20.89 ± 4.46	Class II\1	0.018 × 0.025 SSW (SW MBT) NiTi CLS (150 gr) from miniscrew (U5-U6)	Every 28 days	CR on P-side was significantly faster than CG-side until the end of the 2nd month.	- MAL: N/A - PD: N/A - PH: N/A - RS: no differences (*p* > 0.05)	3 months
Bakr et al. (2023) [29]	RCT, split-mouth design	C	14 patients (2 M, 12 F)	20.4 ± 2.5	Class I biprotrusion	0.017 × 0.025 SSW (SW MBT) NiTi CLS (150 gr) from miniscrew (U5-U6)	Every 2 weeks	No significant difference in the CR between the C-side and CG-side.	- MAL: no differences (*p* > 0.05) - PD: N/A - PH: no differences (*p* > 0.05) - RS: no differences (*p* > 0.05)	3 months
Eid et al. (2024) [30]	RCT, split-mouth design	P	30 patients (16 M, 14 F) SP 15 (9 M, 6 F) MP 15(7 M, 8 F)	15 to 25 SP 17.33 ± 1.88 MP 17.40 ± 1.92	Class I biprotrusion, Class II\1	0.016 × 0.022 SSW (SW Roth) Nitin CLS (150 gr) from miniscrew (U5-U6)	Every 4 weeks	CR registered a significant increase in SG- and MGP-side than CG-side. Non-significant differences have been observed between SP and MP (*p* > 0.05).	- MAL: N/A - PD: N/A - PH: N/A - RS: higher root resorption risk accompanies both SP- and MP-side than CG-side.	3 months
Hawkins et al. (2022) [31]	RCT, split-mouth design	P	20 patients (8 M, 12 F)	18.7 ± 1.12	N/A	0.020 SSW (selfligating), NiTi CLS (250 gr) Nance Transpalatal arch	Every 6 weeks	No statistically significant differences in CR between P-side and CG-side.	- MAL: no differences (*p* > 0.05) - PD: all patients except for one, had minimal pain - PH: N/A - RS: N/A	4,5 months
Jahanbakhshi et al. (2016) [32]	RCT, split-mouth design	C	15 patients (15 F)	25	N/A	0.016 × 0.016 SSW Open loop (200 gr) Miniscrew (U6-U7)	Every 2 weeks	CR is faster on C-side than CG-side (Averages of 1.8 mm/month vs. 1.1 mm/month)	- MAL: N/A - PD: N/A - PH: N/A - RS: N/A	4 months
Raj et al. (2020) [33]	RCT, split-mouth design	P	20 patients (6 M, 14 F)	23.18 ± 1.41	Class II\1	0.016 × 0.022 SSW, NiTi CLS (150 gr) Transpalatal arch	Every 2 weeks	The rate of CR is higher on P-side at all time intervals (*p* < 0.001).	- MAL: N/A - PD: N/A - PH: no differences (*p* > 0.05) - RS: no differences (*p* > 0.05)	7 months
Raza et al. (2021) [34]	RCT, split-mouth design	C	10 patients (N/A)	18 to 25	N/A	0.018 SSW SSW (SW MBT) NiTi CLS (150 gr) Nance Transpalatal arch	Every 4 weeks	There was an overall reduction in the time taken for canine retraction on C-side (5.7 mo) than CG-side (7.1 mo).	- MAL: N/A - PD: was more in C-side than CG- side at 24 h, but similar after one week. - PH: N/A - RS: CG-side exhibit a greater canine root resorption than C-side	8 months
Sharma et al. (2020) [35]	RCT, split-mouth design	C	16 patients (4 M, 12 F)	14 to 25	Class I biprotrusion, Class II\1	0.016 × 0.022 SSW (SW MBT) NiTi CLS (150 gr) From miniscrew (U5-U6)	Every 3 weeks	CR on C-side is significantly greater than CG-side for the initial 2 months.	- MAL: no differences (*p* > 0.05) - PD: mild to moderate pain, discomfort—PH: no differences (*p* > 0.05) and swelling in C-side. - RS: N/A	6 months
Toodehzaeim et al. (2024) [36]	RCT, split-mouth design	C	12 patients (5M, 7F)	15 to 30	Class I biprotrusion Class II\1	0.019 × 0.025 SSW (SW MBT) NiTi CLS (150 gr) Transpalatal arch	Every 2 weeks	CR was significantly greater on the C-side than CG-side (*p* < 0.05).	- MAL: no differences (*p* > 0.05) - PD: no differences (*p* > 0.05) - PH: no differences (*p* > 0.05) - RS: N/A	4 months

Abbreviations: C, corticotomy; CLS, closed coil springs; CTG, corticotomy-treated group; CG, control group; CR, canine retraction; F, female; M, male; MAL, molar anchorage loss; MP, multiple piezocision; N/A, data not currently available; NiTi, nickel titanium; P, piezocision; PD, pain and discomfort; PH, periodontal health; RCT, randomized clinical trial; RS, root resorption; SP, single piezocision; SSW, stainless steel wire; SW, straight wire.

## Data Availability

The data supporting the findings of the present systematic review are available within the article.

## Supplementary Materials

The following supporting information can be downloaded at: https://www.mdpi.com/article/10.3390/dj13020057/s1. Table S1, PRISMA 2020 Checklist; Table S2, Detailed Description of the Surgical Technique.
